# Efficacy of preoperative halo-gravity traction in the treatment of spinal deformity: systematic review and meta-analysis

**DOI:** 10.1097/MS9.0000000000005332

**Published:** 2026-09-25

**Authors:** Ahmed Hamed Amin, Tibyan Noorallah Mohammed, Abdalmalk Saud Nagshabandi, Abdulaziz Omar Alsehemi, Ahmed Ali E Al Abdullah, Albatoul M. Almargan, Batool A. Alshahrani, Hotoon Sulaiman Alshammari, Layan Albraik, Malak AlShebel, Muteb N. Alotaibi, Reem Mohammed Almutairi, Shaima ShamsEldeen KhalfAllah Ahmed, Shahda ShmsEldeen KhlfAlla Ahmed, Yaser Samir Samman

**Affiliations:** a Consultant Orthopedic and Spine Surgeon, Almanaa General Hospital, Dammam, Saudi Arabia; b Medical School of Khartoum University, Khartoum, Sudan; c Department of Surgery, Al Marefaa University, Diriyah, Saudi Arabia; d King Abdulaziz Hospital, Makkah, Saudi Arabia; e Department of orthopaedic, Almoosa Specialist Hospital, Alahsa, Saudi Arabia; f Najran Health Cluster, Najran, Saudi Arabia; g Faculty of Medicine, University of Jeddah, Jeddah, Saudi Arabia; h Department of Preventive Medicine, KKUH, Riyadh, Saudi Arabia; i College of Medicine, Alfaisal University, Riyadh, Saudi Arabia; j Dalhousie University, Halifax, Nova Scotia, Canada; k Department of Orthopaedics, King Faisal Specialist Hospital & Research Center, Riyadh, Saudi Arabia; l Department of Orthopedic Surgery, Qassim Health Cluster, Buraydah, Qassim, Saudi Arabia; m Faculty of Medicine, University of Khartoum, Khartoum, Sudan; n Aliman General Hospital, Riyadh, KSA; o General sciences Department, Al-Rayan National College of Nursing, Al-Rayan National Colleges, Al Madinah Al Munawwarah, Saudi Arabia

**Keywords:** adult spinal deformity, halo-gravity traction, pediatric spinal surgery, pulmonary function, spinal deformities, traction duration

## Abstract

This systematic review and meta-analysis compares the safety and effectiveness of preoperative halo-gravity traction (HGT) in 593 patients with complicated spinal deformity, combining the data of 20 studies from 2000 to 2024. Significant reductions were achieved in coronal (mean reduction of 36.1°, *P* < 0.001) and sagittal Cobb angles (mean reduction of 28.5°, *P* < 0.001), with maximal radiographic changes following 70–80 days of traction (*I*^2^ = 0%). Children’s groups experienced improved corrections compared to adults, likely due to greater spinal plasticity, although heterogeneity within adult groups (*I*^2^ > 90%) prevented generalizability. Pulmonary capacity was uniformly improved, with collective increases in forced vital capacity (0.233 L, *P* < 0.001) and forced expiratory volume in 1 second (0.245%, *P* = 0.001) with no heterogeneity (*I*^2^ = 0%). Complications were pin loosening (16%) and infection (10.7%), which were very heterogeneous among studies (*I*^2^ > 70%), in accordance with variation in surgical technique and postoperative care. Complications were lower in younger patients (<13 years) and with 70–80-day protocols, but rose with longer traction (>80 days). These findings highlight HGT’s role as a primary preoperative adjunct to pediatric deformities but indicate age-related constraints and the need for standardized protocols for complication avoidance in complex or adult conditions. Prospective trials should be the priority of future research to maximize traction techniques and predictors of response.

## Introduction

Severe spinal deformities, such as complex scoliosis, kyphosis, and kyphoscoliosis, often pose a formidable challenge in surgical management due to rigidity, cardiopulmonary compromise, and neurological risks^[^1,2^]^. Recently, preoperative halo-gravity traction (HGT) has gained popularity as an adjunctive treatment to help in correcting the spinal alignment, improving pulmonary function, and decreasing risks related to surgical correction^[^3,4^]^. HGT stretches the spine progressively over time with axial traction and is used for correcting deformities, improving flexibility of the spine, and minimizing complications, particularly in high-risk patients, whether pediatric or adult^[^5,6^]^.

HIGHLIGHTS

- Halo traction significantly reduced both coronal and sagittal Cobb angles in patients with spinal deformities.
- Greater correction in coronal Cobb angle was observed with shorter traction duration (≤40 days).
- Halo traction improved pulmonary function, with a significant increase in forced expiratory volume in 1 second (FEV1) percentage.
- Overall complication rates were low, with neurological deficits (6.8%) and lower limb weakness (5.6%) reported.
- Pin-related complications (loosening and infection) were relatively common but showed high heterogeneity across studies.
- Included studies demonstrated moderate to high methodological quality, with no low-quality studies identified.
- Halo-gravity traction reduced coronal Cobb angle by 36.1° on average (*P* < 0.001).
- Sagittal angle improved 28.5° after 70–80 days of traction.
- Children had better outcomes due to greater spinal flexibility.
- Forced vital capacity and FEV1 improved consistently across included studies.
- Fewer pin complications in younger patients and shorter traction durations.

In spite of its increasing usage, inconsistent evidence supports the effectiveness and safety of preoperative HGT. The results of these studies have been inconsistent, with some series showing significant improvements in Cobb angle correction, pulmonary function, and neurological safety^[^7,8^]^, while others emphasize complications including pin-site infections, cranial nerve palsies, and patient discomfort^[^9,10^]^. This heterogeneity in findings underlines the need for a comprehensive synthesis of the available evidence to inform clinical practice and optimize patient outcomes.

This systematic review and meta-analysis aims to summarize the literature on the updated role of HGT in clinical practice. Specifically, we seek to evaluate the efficacy of HGT on key outcomes such as radiographic measures of Cobb angle correction and spinal flexibility, including functional outcomes like pulmonary function and neurological safety. We will also discuss the rate and types of complications related to HGT and ways to reduce these complications. This review aims to synthesize available evidence in order to assist in clinical decision-making for the optimization of patient outcomes and identification of gaps in literature that need further exploration.

## Methods

### Search strategy and selection criteria

This systematic review and meta-analysis concerning HGT in the treatment of severe spinal deformity is prepared following the Preferred Reporting Items for Systematic Reviews and Meta-Analyses (PRISMA) Statement **(11) (**Supplemental Digital Content Material 1, available at: http://links.lww.com/MS9/B281). The study protocol was registered in the PROSPERO international prospective register of systematic reviews (CRD420251012191).

We searched PubMed, Cochrane, and Scopus comprehensively for relevant studies from 2000 to December 2024, using the combined text and MeSH terms: “halo-gravity traction” or “halo-traction” or “gravity traction” and “kyphosis” or “kyphoscoliosis” or “scoliosis” or “spine deformity.” English language restriction was used. Study titles and abstracts were reviewed by two independent investigators, and relevant studies were full-text assessed according to the inclusion and exclusion criteria defined below. Disagreements were resolved by a third investigator Figure 1.

**Figure 1.**
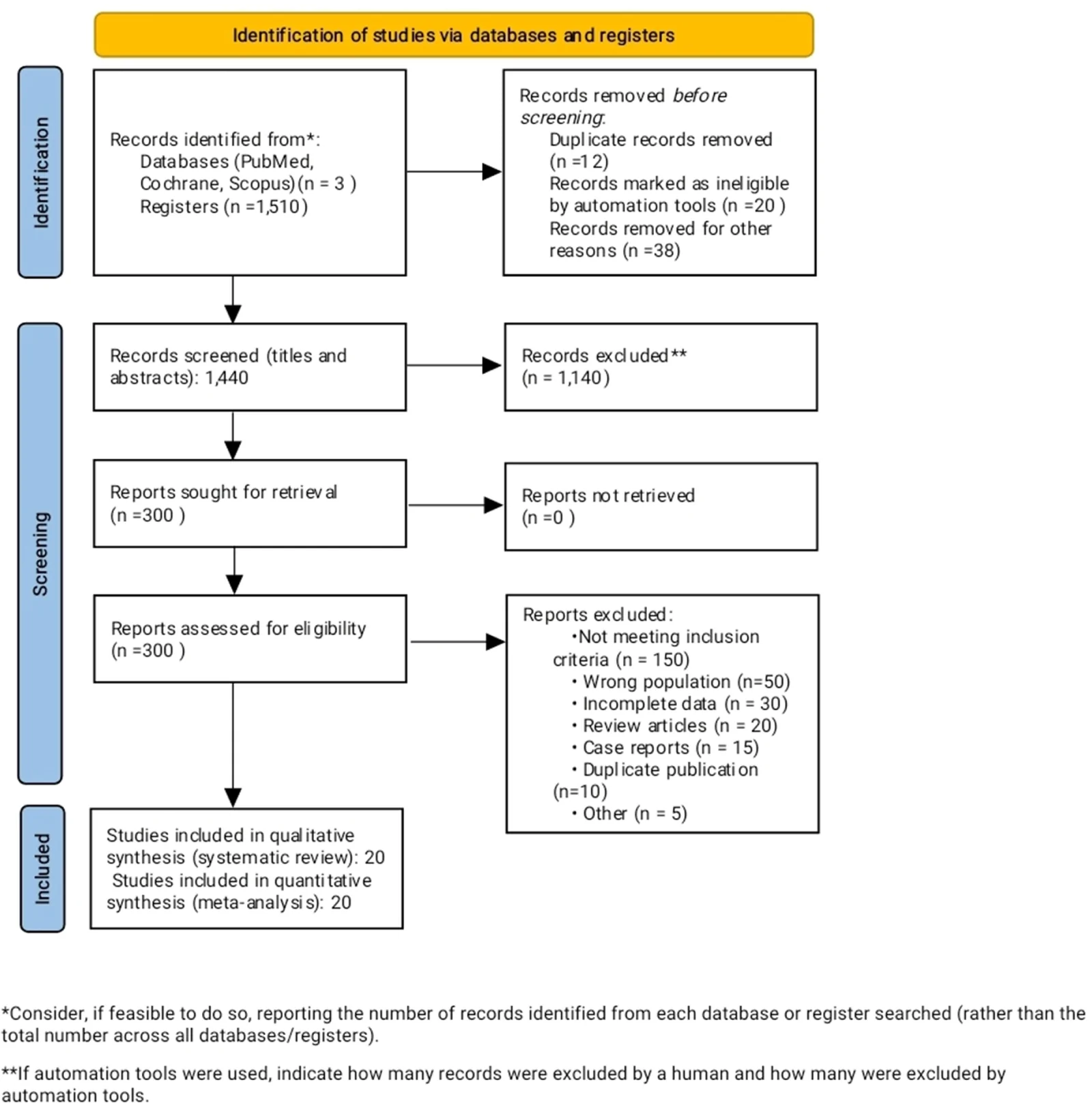
PRISMA flow chart for the review.

### Study selection

We regarded studies as eligible for the inclusions: (1) target patients: patients with severe spinal deformity; (2) intervention: preoperative HGT at least 4 weeks; (3) outcomes: radiographic parameters (coronal and sagittal Cobb angle, height of spine, coronal balance), pulmonary function [forced vital capacity (FVC) %], and nutritional status (BMI). (4) Article types: peer-reviewed studies involving human participants, including observational study designs (e.g., retrospective cohort studies and case series). Reviews and single-patient case reports were excluded; duplicate or multiple publications of the same research were also excluded. (5) Language restriction: articles written in English.

### Data extraction and assessment of study quality

Using a pre specified data abstraction form, two investigators independently extracted the following data from each selected study: (1) study characteristics (author, year of publication, and patient diagnosis, number of patients); (2) HGT characteristics (traction strategy, traction duration, and maximum traction); (3) pre-traction and post-traction radiographic parameters(coronal major cobb angle, sagittal major kyphosis, coronal balance, sagittal balance, the height of spine); and (4) pulmonary function (FVC%) .

Two researchers independently evaluated the quality of the literature according to the PRISMA recommendations. Studies will be evaluated using the Newcastle–Ottawa scale (NOS)^[^11^]^. The NOS consists of nine items, including selection (four points), comparability (two points), and exposure (three points); a study awarded seven or more points was considered high quality. To reduce the influence of subjective factors, the author’s name, institution, journal name, and other related information are hidden during the evaluation process

### Data analysis

Data were analyzed using comprehensive meta-analysis software (CMA) version 3. Outcomes which are found in 3 studies or more were selected for the analysis. Data were presented as mean and standard deviation (SD), in addition to event rates. Two outcomes had missing SD (change in angels’ outcomes), where it was imputed from similar studies in age, design, traction period and sample size. *I*^2^ value was used to assess the heterogeneity of studies, as *I*^2^ >50% (*P* < 0.05) indicates significant heterogeneity. A fixed-effects model was used for all outcomes, unless significant heterogeneity was detected where random effects model was used instead. Subgroup analysis and sensitivity analysis were used to determine the source of heterogeneity. Subgroup analysis was performed based on sample mean age (Adults vs. children) and mean traction duration, as they were the only characteristics reported across all studies. Publication bias was assessed for only one outcome, as it was the only outcome with 10 studies or more (change in Coronal Cobb angle). The bias was presented using Funnel plot and Egger’s test. In all tests, the level of significance used was 0.05.

## Results

### Characteristic of included studies

The total number of included studies was 20. All of them were either retrospective cohort studies or retrospective case series. All of the included studies targeted patients with spinal deformity (scoliosis, kyphosis and kyphoscoliosis) using Halo traction techniques. The number of pins used was variable across the studies, but the maximum traction percentage by body weight was 50%. All studies increased the traction percentage gradually till reaching 50% and also allowed for relief in case of adverse events. The total number of patients was 593, with a mean age of 16.95 years. All studies targeted children except for four studies. The mean traction duration was 64.95 days. Almost all studies targeted patients with thoracic and/or lumbar deformity except one study, which targeted cervical spine deformity^[^12^]^ (Table 1).

**Table 1:** Characteristics of the included studies.

Study	Sample size	Age (mean years)	Traction duration (mean days)	Maximum traction % by body weight	Site of deformity	Overall quality assessment using NOS
Bao *et al*^[^13^]^	21	26.2	76.2	30%	Thoracic Spine	Moderate
Boachie-Adjei *et al*^[^14^]^	13	17.8	110	50%	Thoracic and lumbar spine.	Moderate
Garabekyan *et al*^[^15^]^	21	13	77	50%	Thoracic and lumbar spine.	Moderate
Grabala *et al* ^[^16^]^	20	16.4	36.9	50%	Thoracic and lumbar spine.	Moderate
Iyer *et al* ^[^17^]^	30	9	79	50%	Thoracic and lumbar spine.	Moderate
Iyer *et al*^[^18^]^	96	16.6	97	50%	Thoracic and lumbar spine.	Moderate
Bogunovic *et al*^[^6^]^	33	12.15	70.1	38.5%	N/A	Moderate to high
Bouchoucha *et al*^[^19^]^	15	13.5	64	50%	Thoracic and thoracolumbar spine.	Moderate
Qi *et al*^[^20^]^	30	30	37.6	50%	Thoracic and lumbar spine.	Moderate to high
Qi *et al*^[^21^]^	19	29.7	72.8	41.3%	Thoracic and thoracolumbar	Moderate to high
Rocos *et al*^[^22^]^	24	11.8	42	50%	Thoracic and thoracolumbar spine	Moderate
Verhofste *et al*^[^23^]^	28	11.3	25	29%	Cervical spine	Moderate to high
Caubet and Emans^[^24^]^	12	6.8	82.1	50%	Thoracic and lumbar spine.	Moderate
LaMont *et al*^[^25^]^	107	11.3	82.1	50%	Thoracic and lumbar spine	High
Shi *et al*^[^26^]^	35	14.9	72.3	50%	Thoracic spine	Moderate to high
Shimizu *et al*^[^27^]^	18	33	28.2	50%	Thoracic and lumbar spine	Moderate
Zhang *et al*^[^28^]^	33	17.8	129	40-50%	Thoracic and thoracolumbar spine	High
Shi *et al*^[^29^]^	13	14.9	22.9	30-50%	Thoracic spine	Moderate to high
Nemani *et al*^[^30^]^	14	14	28	50%	Thoracic and thoracolumbar spine	Moderate to high
Li *et al*^[^31^]^	11	18.8	66.85	50%	Thoracic spine	Moderate


N/A, not available.

### Risk of bias assessment

Study quality assessed using the Newcastle–Ottawa Scale (NOS) is presented in the Supplemental Digital Content Material 2, available at: http://links.lww.com/MS9/B282. NOS scores ranged from five to eight stars, indicating overall moderate to high methodological quality. Two studies were rated as high quality (eight stars), nine as moderate-to-high quality (seven stars), and the remaining studies as moderate quality (five to six stars). No study was classified as low quality. Most studies performed well in the selection and outcome/exposure domains, while limited control for confounding factors accounted for lower scores in the comparability domain.

### Change in coronal cobb angle

There were 10 studies that assessed the change in coronal Cobb angle. All studies showed a reduction in the angle after the halotraction. On random effects model, the mean change in coronal Cobb angle was 36.106 degrees (CI: = 30.153–42.059, *P* < 0.001), but the results can’t be generalized due to significant heterogeneity (*I*^2^ = 72.187, *P* < 0.001). Sensitivity analysis was performed, and removal of each study alone didn’t eliminate heterogeneity except Qi *et al*^[^20^]^ study, which yielded *I*^2^ of 44.646 (*P* = 0.071) and pooled estimate of 34.504 (CI: = 31.220–37.77, *P* < 0.001) on a fixed-effect model (Supplemental Digital Content Figure S1, available at: http://links.lww.com/MS9/B283). Publication bias was also assessed, which detected absence of bias in Egger’s regression test (intercept = −2.51, *P* = 0.1626) (Supplemental Digital Content Figure S2, available at: http://links.lww.com/MS9/B284).

Subgroup analysis was performed based on age and traction duration. Regarding traction duration, the subgroups eliminated the heterogeneity, as *I*^2^ value was 0.000 for the 40 days or less group and 34.017 for the more than 40 days group (*P* = 0.516 and 0.157, respectively). In fixed-effects mode, studies with 40 days or less traction duration had significantly higher change in angle compared to the more than 40 days duration (51.477 vs. 33.680 degrees, *P* < 0.001). Subgroup analysis was also performed based on mean age (above 18 years old adults or less than 18 years old children). In the random-effects model, adult studies indicated greater change in coronal Cobb angle than children's studies (36.928 Vs. 35.455 degrees), but it wasn’t significant (*P* = 0.174). The high heterogeneity in the adult group of studies affects the generalizability of the results (*I*^2^ =90.157, *P* < 0.001). However, the children’s group had a pooled estimate of 53.455 (CI: = 30.738–30.393, *P* < 0.001) with absent heterogeneity (*I*^2^ =41.147, *P*-value = 0.117) (Fig. 2).

**Figure 2.**
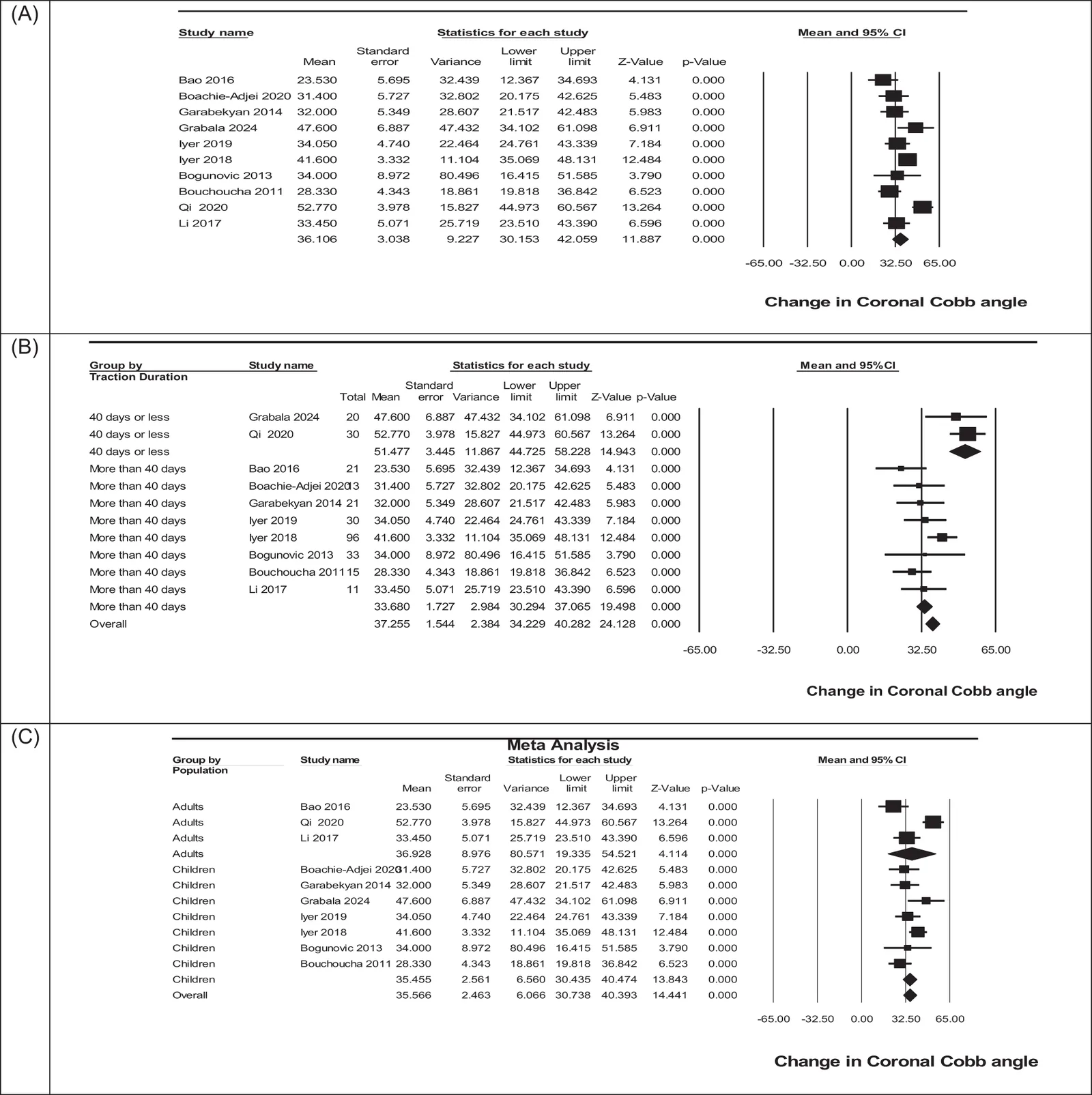
Forest plot for change in coronal Cobb angle, A is pooled plot using random effects model, B is subgroup based on traction duration, and C is subgroup based on age.

### Change in sagittal Cobb angle

There were seven studies that assessed the change in coronal-sagittal angle. All studies showed a reduction in the angle after the halotraction. On random effects model, the mean change in coronal Cobb angle was 28.484 degrees (CI: = 21.297–35.671, *P* < 0.001), but the results can’t be generalized due to significant heterogeneity (*I*^2^ = 72.235, *P* < 0.001). Sensitivity analysis was performed, and removal of each study alone didn’t eliminate heterogeneity (Supplemental Digital Content Figure S3, available at: http://links.lww.com/MS9/B285).

Subgroup analysis was performed based on traction duration alone, as all studies targeted children. Regarding traction duration, the subgroups eliminated the heterogeneity in only two groups, as *I*^2^ value was 0.000 for 70–80 days group and also for the less than 70 days group (*P* = 0.517 and >0.99, respectively). The third group (More than 80 days) showed significant heterogeneity (*I*^2^ = 90.661, *P* = 0.001). The first group (70–80 days) had a significantly greater reduction in the angle (32.754, CI: = 27.947–37.562) compared to other groups (*P* < 0.001) in fixed-effect model. But the presence of only one study in the less than 70 days group and also the high heterogeneity of the more than 80 days group hinder these results (Fig. 3).

**Figure 3.**
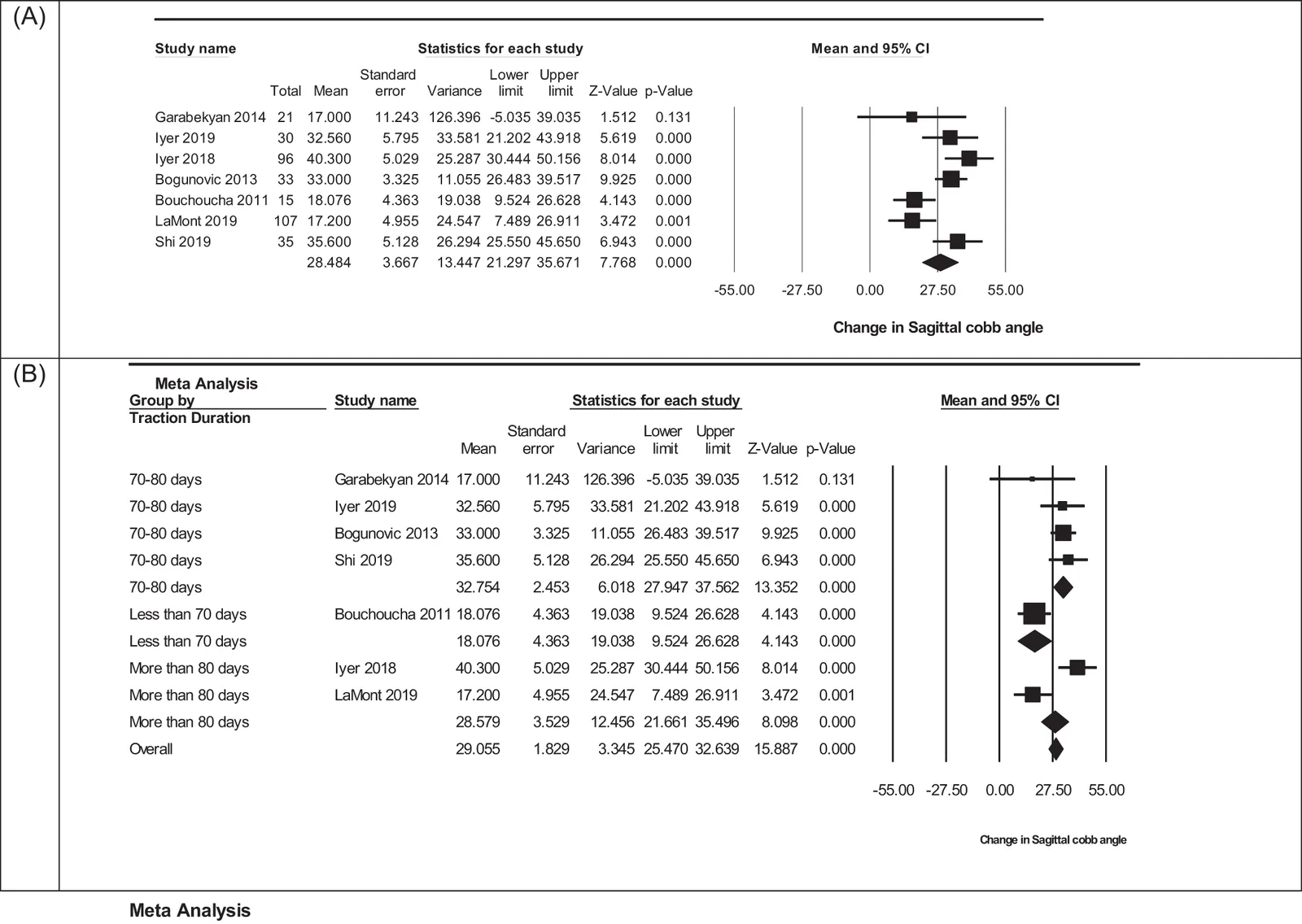
Forest plot for change in sagittal Cobb angle, A is pooled plot using random effects model, B is subgroup based on traction duration.

### Change in forced expiratory volume in 1 second percentage

Three studies addressed the change in forced expiratory volume in 1 second (FEV1) percentage. All studies reported improvement in FEV1%. In the fixed-effect model, the pooled estimate of change in FEV1 was 0.245% (CI: = 0.101–0.389, *P* = 0.001). No heterogeneity was detected (*I*^2^ = 0.000, *P* = 0.907) (Fig. 4).

**Figure 4.**
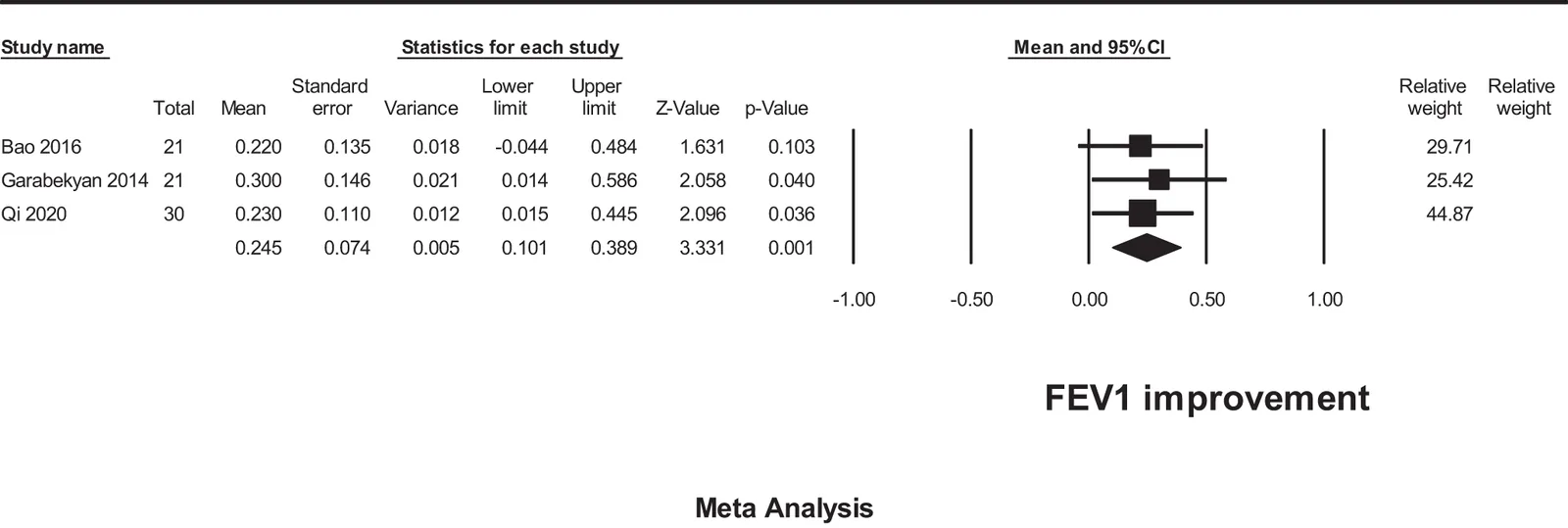
Fixed effect model for change in FEV1.

### Change in FVC

Three studies addressed the change in FVC percentage. All studies reported improvement in FEV1 %. In the fixed-effect model, the pooled estimate of change in FVC was 0.233 L (CI: = 0.196–0.270, *P* < 0.001). No heterogeneity was detected (*I*^2^ = 0.000, *P* = 0.829) (Fig. 5).

**Figure 5.**
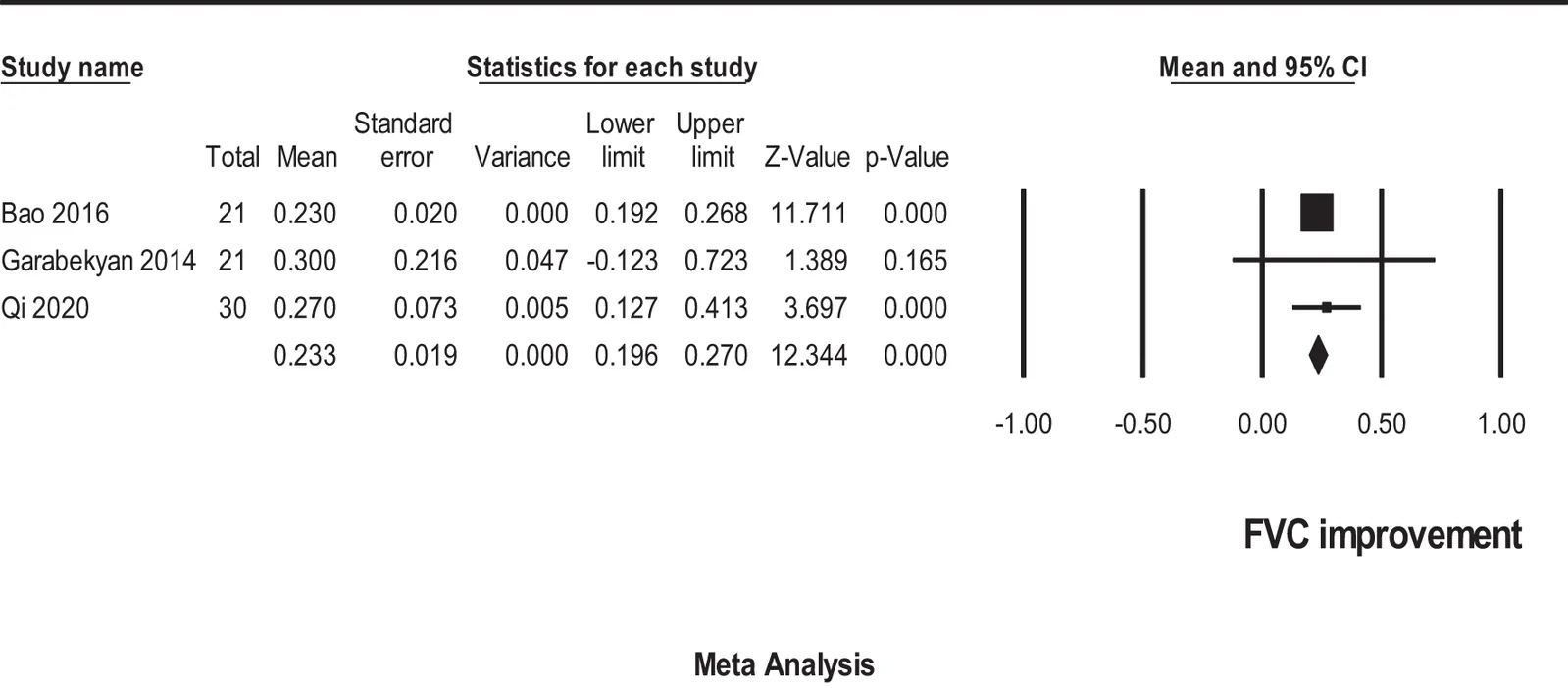
Fixed effect model for change in FVC.

### Adverse events

Multiple adverse events were reported across studies. Lower-extremity weakness was reported in 4 studies, while neurological deficits were reported in 3. In the fixed-effect model, the pooled estimate of lower-extremity weakness event rates was 0.056, indicating a percentage of 5.6% (CI: = 0.021–0.139, *P* < 0.001). No heterogeneity was detected (*I*^2^ = 0.000, *P* = 0.992) (Fig. 6). Regarding neurological deficit, the pooled event rate in fixed-effect model was 0.068, indicating a percentage of 6.8% (CI: = 0.028–0.154, *P* < 0.001). No heterogeneity was detected (*I*^2^ = 0.000, *P* = 0.581) (Fig. 7).

**Figure 6.**
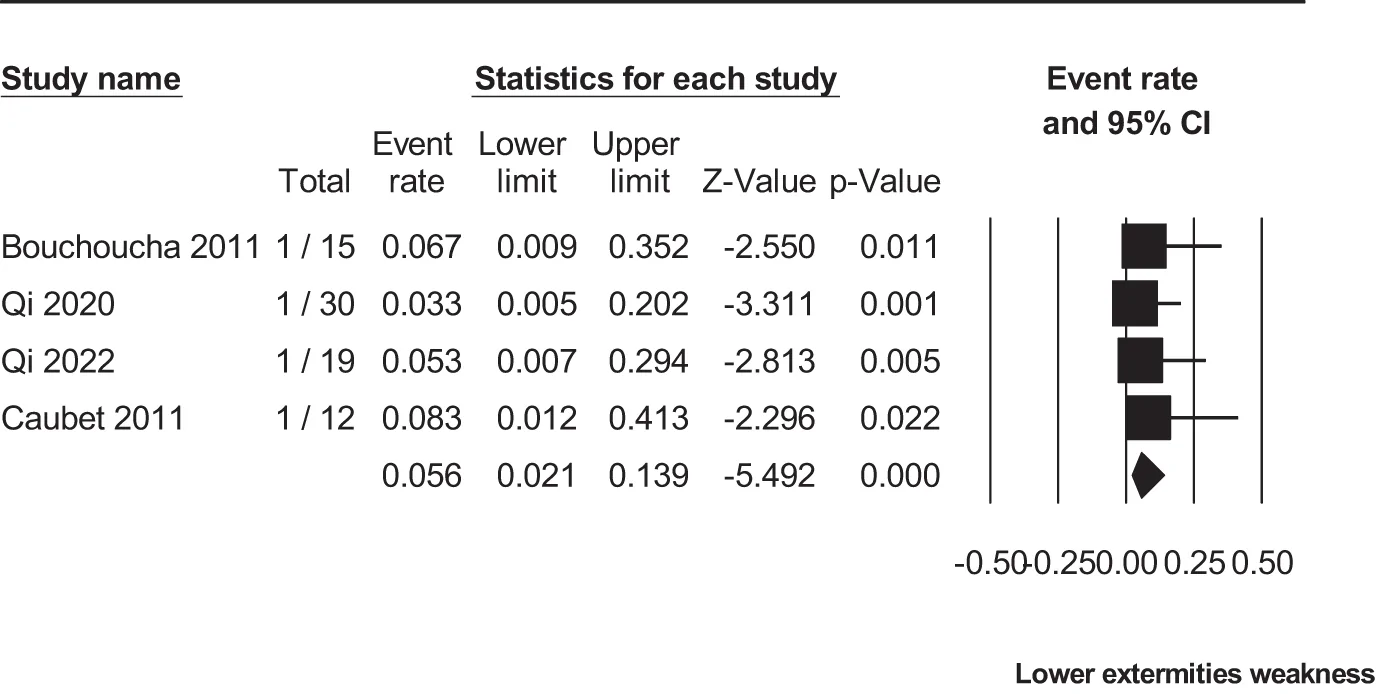
Fixed effects model for lower extremities weakness.

**Figure 7.**
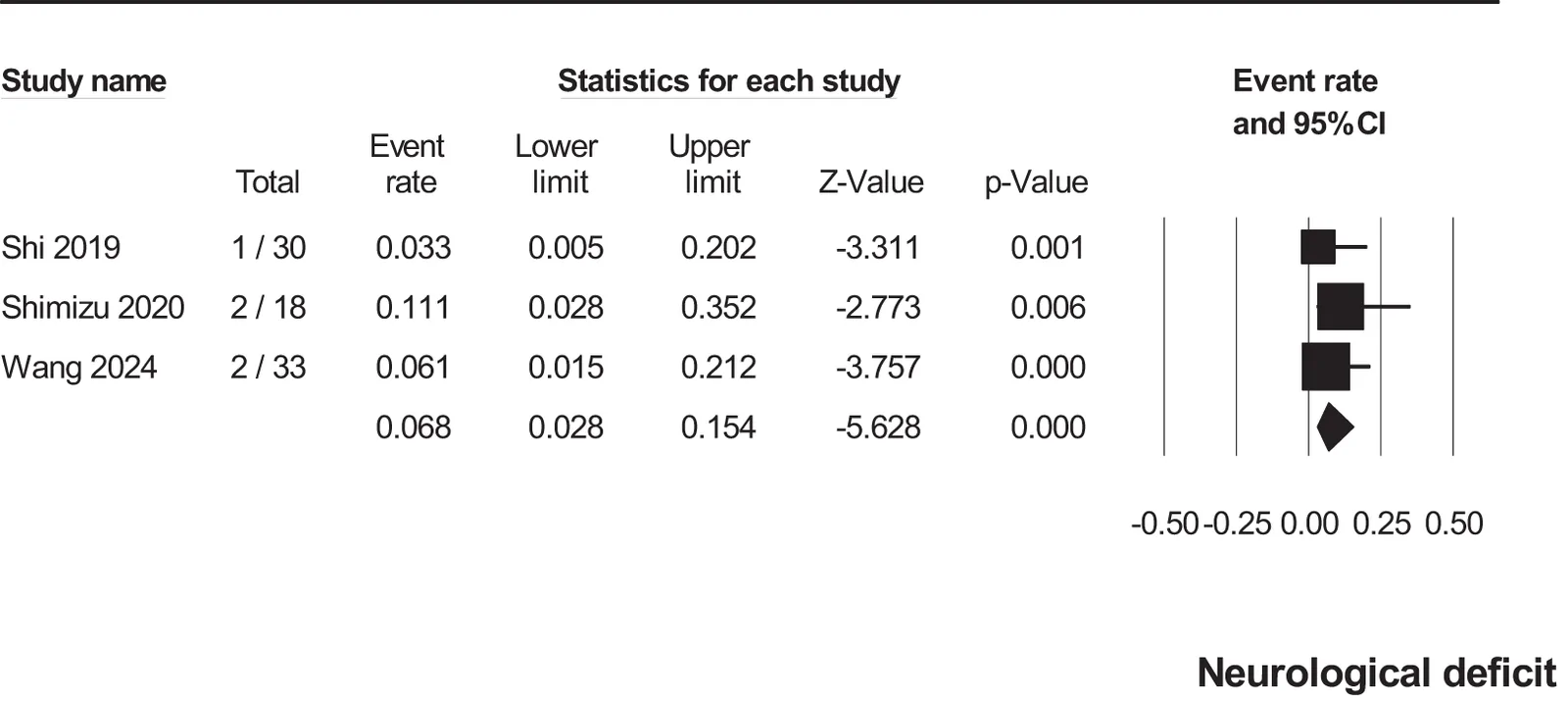
Fixed effects model for neurological deficits.

Pin-associated events also occurred across the studies, with pin loosening being reported in six studies, while pin-associated infections were reported in seven studies. On random effects model, the pooled event rate of pin loosening was 0.160 indicating 16% (CI: = 0.061–0.361, *P* = 0.003), but the high significant heterogeneity hinder the generalizability of this result (*I*^2^ =74.644, *P* < 0.001) (Supplemental Digital Content Figure S4, available at: http://links.lww.com/MS9/B286). While pin-site infection had a pooled event rate of 0.107, indicating a percentage of 10.7% (CI = 0.045–0.232, *P* < 0.001), but the high heterogeneity hinder this result also (*I*^2^ = 79.005, *P* < 0.001) (Supplemental Digital Content Figure S5, available at: http://links.lww.com/MS9/B287). Sensitivity analysis was performed for both outcomes, and removal of each study alone didn’t eliminate heterogeneity for both of them (Supplemental Digital Content Figure S6, available at: http://links.lww.com/MS9/B288) (Supplemental Digital Content Figure S7, available at: http://links.lww.com/MS9/B289).

Subgroup analysis was performed based on age and traction duration. Regarding traction duration, the subgroups didn’t fully eliminate the heterogeneity as *I*^2^ value was 0.000 for 70–80 days group, 0.000 for More than 80 days group and 72.463 for less than 70 days group (*P* = 0.661, >0.99 and 0.057, respectively). In fixed-effect mode, studies with 70–80 days of traction duration had a significantly lower event rate of pin loosening (0.063, CI: = 0.024–0.156) compared to the other groups (*P* < 0.001). But the presence of only one study in the more than 80 days group and the high heterogeneity of the less than 70 days group hinder this result. Subgroup analysis was also performed based on mean age (above 18 years old-adults- or less than 18 years old children). In the random-effects model, the pooled event rate in children studies was 0.187, indicating a percentage of 18.7% for pin loosening (CI: = 0.068–0.422, *P* = 0.013), but this result was hindered by high heterogeneity in this group (*I*^2^ = 76.507, *P* = 0.002). The adults group included only one study^[^21^]^ (Supplemental Digital Content Figure S4, available at: http://links.lww.com/MS9/B286). Regarding pin-site infection, subgroups were also performed, but they couldn’t fully eliminate the heterogeneity. 70–80 days group had a significant lower event rate in fixed-effect model (0.073, CI: = 0.033–0.152) compared to other groups (*P* = 0.004), but the high heterogeneity in more than 80 days group hinder this result (*I*^2^ = 92.127, *P* < 0.001). Age subgroups also didn’t fully eliminate heterogeneity. Children below 13 years old group showed significantly lower event rate of infections on fixed effects model (0.043, CI: = 0.018–0.100) than other groups (*P* < 0.001), but the high heterogeneity in teenager group (13–18 years old) hinder this result (*I*^2^ =55.794, *P* = 0.104) (Supplemental Digital Content Figure S5, available at: http://links.lww.com/MS9/B287).

## Discussion

This meta-analysis and systematic review demonstrate that preoperative HGT successfully reduces spinal deformities, with the most pronounced corrections in coronal and sagittal Cobb angles achieved in 70–80 days of traction, in favor of its ongoing role in preoperative optimization. In the adult population, however, the efficacy of HGT remains compromised by increased spinal rigidity, and traction durations exceeding 80 days may not provide additional benefits while possibly increasing the complication risk.

Our findings confirm that HGT achieves significant coronal and sagittal Cobb angle corrections, with greater improvements in younger patients. Pediatric age groups had greater flexibility and traction response, consistent with findings from Nunes *et al*^[^32^]^. Adult patients showed very high heterogeneity (*I*^2^ > 90%), which limited generalizability. This agrees with Liu *et al*^[^33^]^, who established that skeletal maturity significantly reduces HGT’s ability to correct. These results show that HGT should receive priority in pediatric deformity, while other techniques, such as vertebral column resection, can be utilized to correct rigid adult deformities.

Subgrouping by traction duration showed that the 70–80-day protocol was linked with best correction and minimal heterogeneity. This again confirms that traction for more than this period does not improve results and may be complicated, as also found by Park *et al*^[^34^]^. Traction durations of less than 40 days were also linked with significantly greater angular corrections, which can either be a result of larger initial traction weights or patient selection bias.

HGT was associated with long-term improvement in pulmonary function, as evidenced by the improvement in FVC by a mean of 0.233 L (*P* < 0.001) and in FEV1 by 0.245% (*P* = 0.001). The absence of heterogeneity between studies lends further strength to the validity of these findings. These results are in accordance with Yang *et al*^[^3^]^, who demonstrated that HGT enlarges lung volume through the decrease of thoracic compression and the increase of ribcage excursion. Given that pulmonary function is one of the most significant predictors of postoperative recovery, this also provides a rationale for the application of HGT in preoperative optimization, especially in individuals with thoracic scoliosis and restrictive lung disease.

HGT complications are very common but are extremely heterogeneous between studies. Neurological complications such as lower extremity weakness and deficits were reported consistently with low heterogeneity, suggesting that these are expected risks from mechanical stress or transient nerve compression. This is in agreement with Pham *et al*^[^35^]^, where similar rates of temporary neurological symptoms with traction were found. On the other hand, pin-related complications (loosening: 16%, infection: 10.7%) were subject to high heterogeneity, which mirrors variations in surgical technique, post-traction care, and patient demographics. These results align with Popescu *et al*^[^36^]^, in which infection rates are correlated with pin-care protocols and traction duration. Subgroup analysis implicated traction duration 70–80 days and younger patient age (<13 years) with fewer complications. Particularly, pin loosening was minimal in the 70–80-day group (6.3%), whereas infection rates were considerably lower in children <13 years (4.3%) but higher in teenagers, similar findings to Verhofste *et al*^[^23^]^. These results point to the necessity of age-specific traction protocols and the standardization of pin-care protocols to reduce infection risks.

The substantial heterogeneity observed across several outcomes, such as the coronal and sagittal Cobb angle correction and pin-related complications, requires careful interpretation. This variability is likely multifactorial, reflecting differences in traction protocols and duration, patient selection (especially age), and additional sources of heterogeneity may include differences in surgical strategies following traction, and institutional variations in pin insertion techniques and pin-care protocols. Consequently, pooled estimates derived from analyses with high heterogeneity or limited study numbers should be interpreted with caution, as they may not be generalizable across all patient populations or clinical settings.

Also, the interpretation of these findings should consider the methodological quality and risk of bias of the included studies. Although most studies were rated as moderate to high quality, comparability scores were frequently limited due to inadequate adjustment for confounding variables such as baseline deformity severity, skeletal maturity, and comorbidities. This limits causal inference and suggests that the observed associations should be interpreted with caution.

Future studies need to try to standardize traction time guidelines, determine the optimum threshold for various patient populations, and find out long-term results to determine whether preoperative HGT reduces postoperative complications. Being able to determine factors such as skeletal maturity, neuromonitoring techniques, and pre-traction spinal stiffness can foretell HGT failure or success. Lastly, streamlining pin-care protocols with antimicrobial precautions and fixation techniques can reduce infection and loosening rates.

## Conclusion

Preoperative HGT appears to be a beneficial adjunct in pediatric patients with severe spinal deformities, achieving meaningful Cobb angle correction and demonstrating potential improvement in pulmonary function. However, pulmonary outcomes should be interpreted cautiously, as these findings are based on a limited number of studies. In adult patients, the effectiveness of HGT remains uncertain due to very high heterogeneity across studies, which limits the strength and generalizability of pooled estimates. Traction durations exceeding 80 days do not appear to be beneficial. Standardized protocols, particularly in pin-care and traction duration, are necessary to minimize complications and improve patient outcomes. Overall, these findings highlight the need for standardized traction protocols and well-designed prospective studies to better define optimal patient selection, traction duration, and strategies to minimize complications and improve outcomes across heterogeneous patient populations.

## Data Availability

All data analyzed in this systematic review and meta-analysis were obtained from previously published studies. No original datasets were generated during the current study. The data supporting the findings of this study are available within the published articles included in the review and the supplementary materials.

## Research registration unique identifying number (UIN)

The study protocol was registered in the PROSPERO international prospective register of systematic reviews (CRD420251012191).
